# Moving Focus from Weight to Health. What Are the Components Used in Interventions to Improve Cardiovascular Health in Children?

**DOI:** 10.1371/journal.pone.0135115

**Published:** 2015-08-11

**Authors:** Claire Friedemann Smith, Carl Heneghan, Alison Ward

**Affiliations:** 1 Health Behaviour Research Center, Department of Epidemiology and Public Health, University College London, London, United Kingdom; 2 Department of Primary Care Health Sciences, University of Oxford, Oxford, United Kingdom; University of Calgary, CANADA

## Abstract

**Introduction:**

Obesity in childhood impacts on many areas of the child’s current and future health, including their cardiovascular health. To date many attempts have been made to design interventions to tackle excess childhood weight but with limited success. We aimed to establish the components common to interventions in children that improve cardiovascular health parameters.

**Methods:**

We searched the following databases: EMBASE 1974-week 3 November 2014, Ovid Medline 1946 Present, and PsychINFO 1967-Present for studies reporting interventions in healthy young people under the age of 18. Included interventions had to contain an education component and have been carried out in a community, school, or clinical setting. Papers had to report on at least one of the pre-specified CVD risk parameters and at least one non-biological outcome from knowledge, attitudes or behaviours.

**Results:**

We retrieved 2451 papers, from which 12 studies (18 papers) of 3046 participants were included. From the selected papers we identified four component themes; Health Behaviours, Self-Concept, Practical and Cognitive Tools, and Intervention Characteristics. The subcomponents that made up these themes were fairly consistent across the studies analysed although the studies varied in their duration, settings and children with which they were carried out. Nine of the studies were able to bring about positive change in at least one biological and one non-biological aspect of child cardiovascular health.

**Conclusion:**

The component themes identified here were common to intervention studies that had success in improving parameters of cardiovascular health. We suggest that the focus of childhood health interventions be moved from weight onto cardiovascular health parameters and that future interventions use the lessons learned by their predecessors to incorporate those components that are associated with successful interventions.

## Introduction

Worldwide trends show that the numbers of children who are overweight or obese has increased dramatically over recent years: over a third of the child population in many countries are classed as such [1, 2]. While in the past excess weight was a condition primarily associated with more prosperous nations, obesity is now a global health problem and the second largest contributor to global disease burden [3, 4].

Obesity in the young could have wide reaching implications for health and for society. Obesity is associated with numerous physical and mental health comorbidities [1], as well as functional change to the cardiovascular system [5–8], and detrimental effects on society through lost productivity and financial burdens on health services [9, 10]. In addition, children with obesity face discrimination and stigma due to their weight status that also has negative consequences for both their physical and mental health. A review of the literature described the impact that weight bias can have on overweight children and adolescents, and detailed damaging effects on self-esteem, social interactions and even physical health through disordered eating and physical activity habits [11]. Additionally reports of increased blood pressure in those who experience discrimination because of their physical appearance were detailed, further highlighting the need to tackle this problem, but only through interventions that do not reinforce any existing weight stigma [11].

In response to this growing problem, the research community has designed and tested a multitude of behaviour change interventions to reduce weight and improve the health outcomes of children who are overweight and obese. The approaches used have been varied and have attempted to improve outcomes by targeting particular age groups [12, 13], specific health-related behaviours [14–16], and individual cultures [17]. Despite these attempts, shortcomings pervade this area of research. Systematic reviews of interventions have reported substantial limitations including: non comparable groups at baseline, lack of high quality trials, high amounts of heterogeneity leading to small and non-significant effects, effects which are not sustained at follow-up, and studies that are poorly reported [13–18].

The components of the studies summarised by the reviews above, such as dietary education, increasing moderate to vigorous physical activity, and parent education, are some of the most widely used and reported but form only a small part of the total variation of intervention components. These reviews were also all carried out with studies looking either exclusively or at least primarily at the effect of the intervention on weight-related outcomes such as weight loss, BMI reduction, or a reduction in the prevalence of being overweight. Our component analysis was performed as part of the wider WE ♥ Health study in which the aim was to move focus away from weight loss and on to cardiovascular health and in doing so highlight the importance of a healthy body over a slim body. By learning from the successes and failures of previous interventions, it was intended that future interventions could incorporate these lessons learned and build on the existing literature. As such, our aim was to identify the aspects of interventions that most frequently led to improvements in child cardiovascular health outcomes.

## Methods

### Ethics Statement

Ethical approval was not required as this is a synthesis of previously published research. The authors received no specific funding for this study and declare no conflicts of interest.

### Component analysis

This component analysis was methodologically similar to a previous paper that analysed the components of self-monitoring interventions for CVD [19] and was carried out in six stages:

1. A literature search for relevant interventions.
2. Description of the included interventions.
3. Extraction of the intervention components into a pre-defined template.
4. Extraction of further information about the included interventions such as theoretical basis and the results achieved.
5. Authors contacted to provide missing information if any of the previous stages are not able to be completed from the papers.
6. Finally the extracted information was examined for components that were most frequently associated with success in interventions.

**Fig 1:**
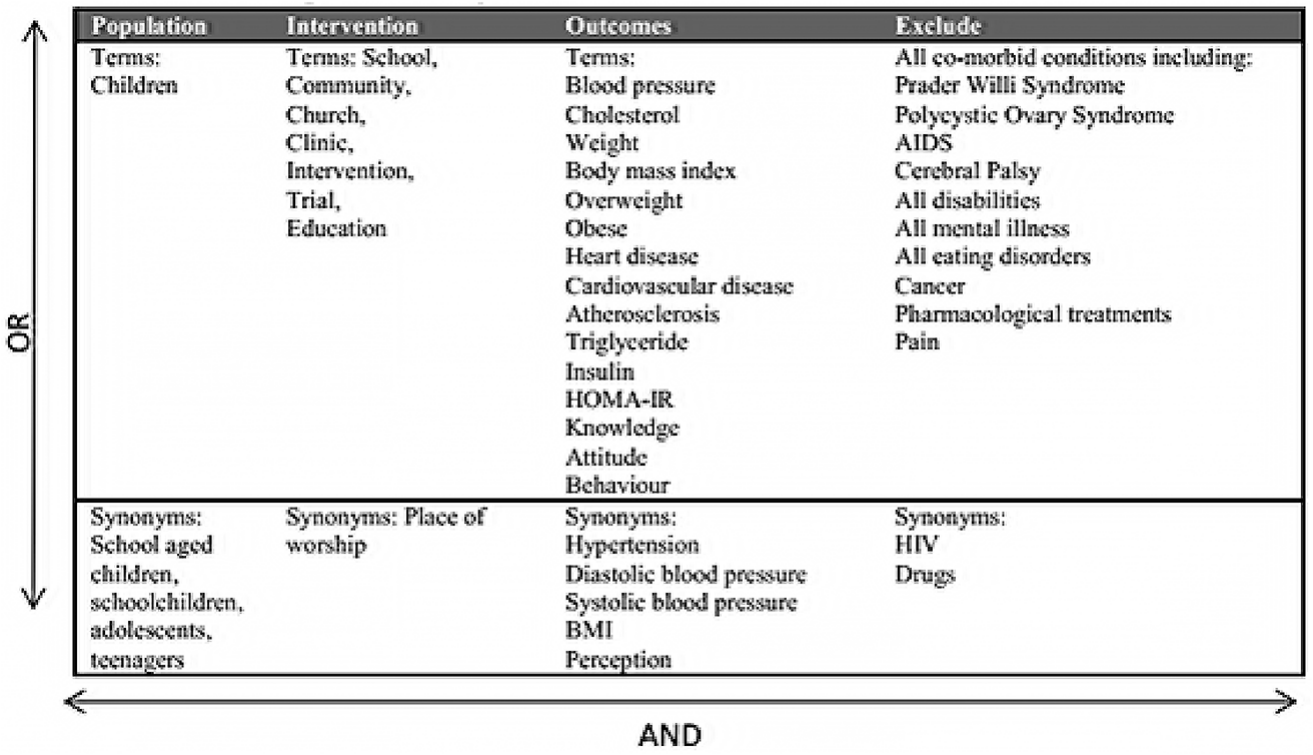
Search terms.

We searched the following databases: EMBASE 1974-week 3 November 2014, Ovid Medline 1946 Present, and PsychINFO 1967-Present. The search terms detailed in Fig 1 were truncated with wild card characters where necessary and searched in each of the databases. The PRISMA Checklist for this study can be seen in S1 PRISMA Checklist.

Studies included in the component analysis had to report on an intervention carried out in healthy young people under the age of 18 and use a control group so that the likelihood of any results occurring due to the effect of the intervention could be judged. We defined an improvement or healthy change in the variables of interest as a change in the intervention group compared to the control group, that could either be an increase or decrease depending on the variable, which reached a significance level of p≤0.05 as reported by the study. Included interventions had to contain an education component and had to have been carried out in a community, school, or clinical setting. Papers had to report on at least one of the CVD risk parameters searched as an outcome as well as the change in at least one non-biological outcome from knowledge, attitudes or behaviours. The inclusion criteria are summarised below:

- Intervention studies.
- Must have a control group.
- Must be group interventions.
- Interventions must be carried out in a school, community or clinical setting.
- The intervention must have an education component.
- Must measure at least one CVD risk parameters searched.
- Must measure change in at least one of knowledge, attitudes or behaviours.

Studies were excluded if they contained a pharmacological element or if the paper did not report on changes in both the CVD risk parameters and the change in the knowledge, attitudes, or behaviours of the children. Papers were not excluded based on country of origin or date or language of publication. The exclusion criteria are summarised below:

- Use of a pharmacological treatment.
- Participants have a known co-morbid condition.
- Failure to report sufficient outcome data.

## Results

**Fig 2:**
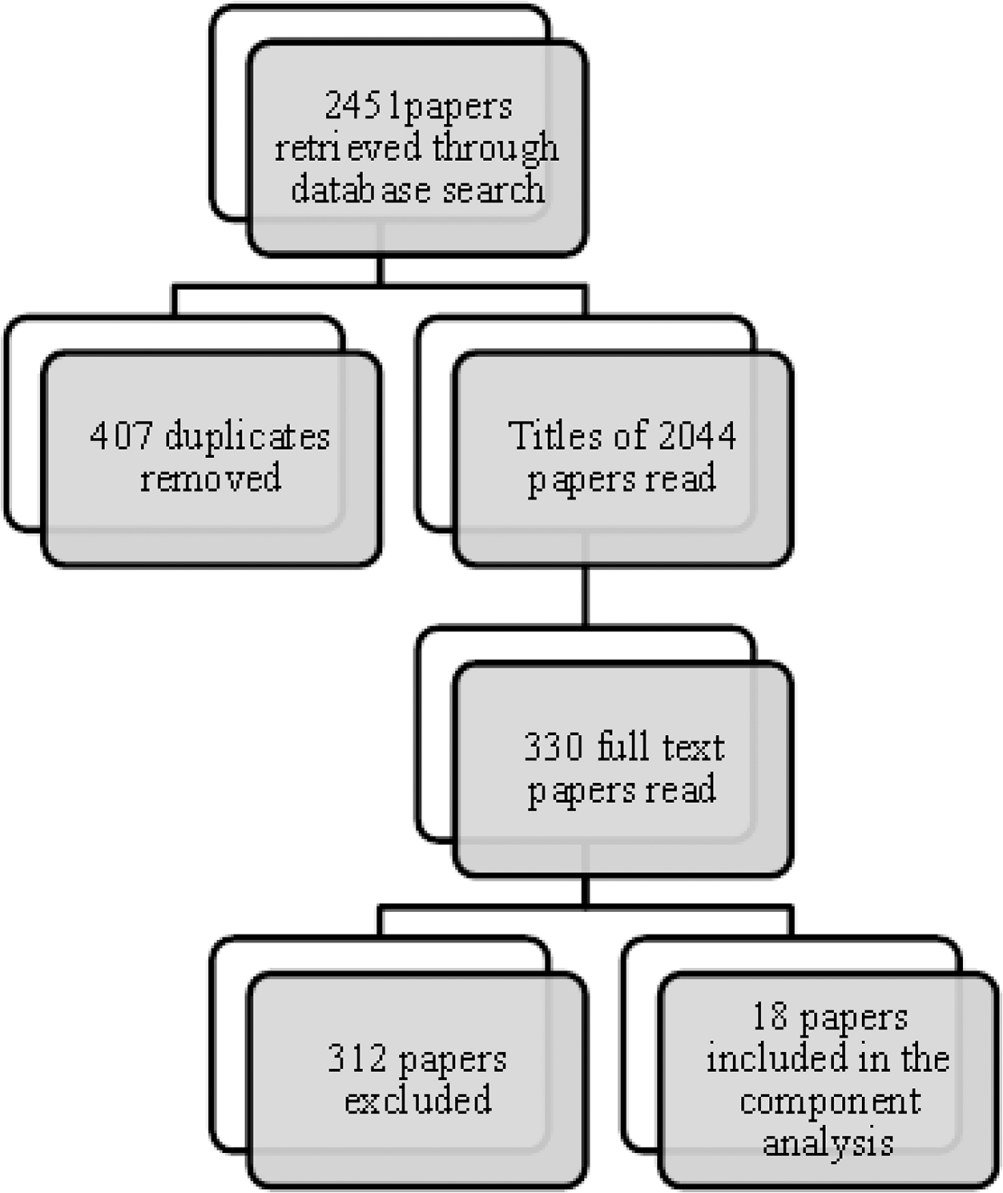
Selection process of papers included in the component analysis.

The database search retrieved 2451 papers, which were de-duplicated to leave 2044 papers. The titles of these papers were read and any that did not meet the inclusion criteria were excluded. The full text of the remaining 330 papers were read and excluded if any exclusion criteria were met. The most common reasons for exclusion from the component analysis were a lack of control group and a failure to report changes to CVD risk parameters as well as knowledge, attitudes or behaviours. Finally 12 studies (18 papers) which met all of the inclusion criteria and reported their data in sufficient detail were selected (Fig 2).

Table 1 shows the main characteristics of the included studies. The studies described interventions that lasted between six weeks and two years and where maintenance of the results was measured, these had follow-up periods of between six weeks and one year post intervention. A number of the studies were based on theoretical models such as the Theory of Planned Behaviour [20], Cognitive Behavioural Therapy [21–26], Social Cognitive Theory [22–27], and Cognitive Theory [28–30].

**Table 1:** Characteristics of included studies.

Author	Study name	Country of origin	Number of participants	Age	Setting	Design	Duration	Outcomes measured	Achieved improvements in biological and non-biological outcomes
Taylor et al. (2007) [33,34]	APPLE	New Zealand	730	6–9 years	School	Non-randomised, controlled physical activity, nutrition and educational intervention	2 years	Height, weight, waist circumference, pulse rate, blood pressure, BMI. Dietary intake questionnaire, physical activity through accelerometry, physical activity questionnaire, time spent television	Yes
Angelopoulos et al. (2009) [20]	CHILDREN	Greece	646	9–10 years	School	Cluster randomised, controlled nutrition and physical activity intervention	1 year		Yes
Melnyk et al. (2009) [21]	COPE TEEN	USA	19	14–15 years	School	Cluster randomised, controlled educational intervention	9 weeks		Yes
Schofield et al. (2005) [38]	GSOP	Australia	85 girls	15 years	School	Non-randomised, controlled physical activity self-monitoring and education intervention	6 weeks, 6 week follow-up		No
Stock et al. (2007) [35]	Healthy Buddies	Canada	383	4–11 years	School	Non-randomised, controlled health promotion intervention	21 weeks		Yes
Johnson et al. (1991) [37]	Heart Smart Program	USA	19 children 23 parents	9–13 years	School	Non-randomised, controlled education, physical activity and nutrition intervention involving parents	12 weeks		No
Nguyen et al. (2012) [22,23,24,25,26]	LOOZIT	Australia	151	13–16 years	Local community health centre and children’s hospital	Randomised, controlled lifestyle modification intervention	7 weeks, 24 month follow-up		Yes
Sacher et al. (2010) [27]	MEND	UK	116	9–11	Community (e.g. schools and sports centres)	Randomised, controlled healthy lifestyle intervention	9 weeks, 12 week follow-up		Yes
Bayne-Smith et al. (2004) [36]	PATH	USA	442 girls	14–17 years	School	Randomised, controlled heart health education and physical activity intervention	12 weeks		Yes
Nemet et al. (2005) [32]	-	Israel	46	8–14 years	Child health and sports centre, Meir General Hospital, Tel Aviv University	Randomised, controlled diet, behaviour and physical activity intervention	3 months, 1 year follow-up		Yes
Park et al. (2007) [31]	-	Korea	44	13–15 years	School	Randomised, controlled lifestyle and education and exercise intervention	12 weeks		No
Tershakovec et al. (1998) [28–30]	-	USA	342	6 years	Home	Randomised, controlled nutrition education intervention	10 weeks, 12 months follow-up		Yes

The studies reported some improvements in the outcomes of interest following the interventions. The weight of the intervention group was improved in four studies [31–35] while reductions in BMI and BMIz score were seen in seven studies [20, 22–27, 31–35]. The biochemical and functional parameters of CVD were improved by some of the interventions. Specifically, systolic blood pressure was improved in five studies [20, 27, 31, 35, 36], and diastolic blood pressure in two studies [20, 36]. Improvements were also seen in the children’s high density lipoprotein (HDL) cholesterol in one study [21], low density lipoprotein (LDL) cholesterol in two studies [28–31], total cholesterol in two studies [22–26, 31], triglycerides in two studies [22–26, 31], and insulin resistance measured by the homeostasis model assessment of insulin resistance (HOMA-IR) in one study [31] (see S1 Table).

The non-biological outcomes also saw some improvements after the interventions. Health behaviours were improved in eleven studies [20–30, 32–38], health knowledge was increased in four studies [21, 28–30, 35, 36], and the children’s attitudes to health were improved in one study [35]. Table 2 shows the outcomes that were improved in each of the studies.

**Table 2:** Component themes.

Main theme	Sub-components
**Health Behaviours**	Diet, physical activity, lifestyle compatible activities, focus on fitness, familiarity with fruit and vegetables
**Self-Concept**	Self-esteem, body image, self-efficacy
**Practical and Cognitive Tools**	Goal setting, problem solving, stress management, practical skills (e.g. interpreting nutrition labels and food preparation)
**Intervention Characteristics**	Take home materials, deliverer resources, focus away from weight, involvement of others (e.g. parents, peers, community), interactive, homework, focus on behaviour, attitude and knowledge change, professional involvement (e.g. dietician).

The included studies were not without shortcomings in their methods and data. The most common of these being improvements that were not sustained at follow-up, short follow-up periods, and when confounding factors were controlled for, changes seen in only one age or gender group. The inclusion of a follow-up period was an area in which much variation was seen between studies. Of the 12 included studies, five had follow-up periods ranging from six weeks to one year (Table 1). It may be expected that studies that followed-up their participants would have been less likely to achieve improvements in the biological and non-biological factors of interest, as the effect of the study lessens over time. This was not the case, however, as of the five studies only one, which also had the shortest follow-up, did not report improvements in both the biological and non-biological factors.

### Components common to changes in outcomes

In order to ascertain which components were present in studies that brought about a positive change in the outcomes of interest, any papers which failed to improve at least one biological and one non-biological outcome were excluded. This left nine out of the twelve included studies remaining [20–30, 32–36]. Of the three excluded studies, Schofield, Mummery and Schofield (2005) [38] and Johnson et al. (1991) [37] were able to bring about some significant improvements in physical activity and dietary measures, however these were not accompanied by significant improvements in the biological factors of interest as defined above. Park et al. (2007) [31] reported significant improvements in many of the biological factors of interest, but did not report any changes in non-biological factors. These studies reported interventions that were among the shortest of those included in this analysis which could have meant that they were not long enough for significant improvements to be seen. There were those, however, that did achieve improvements in both the biological and non-biological factors that were of similar length, and so we must assume that the different outcomes seen were due to the differences in how the interventions were constructed, delivered, and assessed. For the details of which factors were improved in all 12 studies see S1 Table.

Table 2 shows the components common to the nine studies that had achieved an improvement in both biological and non-biological outcomes. The components were grouped into component themes and each theme is discussed below.

### Health behaviours

The subcomponents of this theme focussed on instructing the children in healthy eating [20–30, 32–34, 36] and physical activity [20–26, 32–36]. The healthy eating component included learning about the food pyramid [32], setting targets like reducing the consumption of sugar sweetened drinks, increasing the consumption of fruit and vegetables [27, 33, 34], and increasing the children’s familiarity with different fruit and vegetables [20]. Physical activity included encouraging the children to increase the amount of time they spent being active [32], physical education (PE) lessons that placed greater importance on fitness [20, 32, 35], and activities that could easily be incorporated into the children’s lifestyles [21, 33, 34]. Of the studies that included instruction in healthy eating, all nine studies brought about significant (p-values ≤ 0.05) improvements in the children’s diets. Such improvements included reductions in carbonated and fruit juice drinks, reduction in fats and oils consumed, increases in the number of children eating breakfast and increases in fruit and vegetable servings consumed. Seven of the eight studies targeting physical activity brought about significant (p-values ≤ 0.05) improvements which included reductions in screen time and increases in extra-curricular physical activity.

### Self-concept

This theme included three sub-components; self-esteem, healthy body image [20–26, 35], and self-efficacy [20]. Self-esteem and healthy body image was addressed through discussions to change self-concept, role-modelling, education in growth and development, and introducing techniques such as positive thinking and self-talk. Self-esteem and body image were assessed using a pictorial current-self and ideal-self rating scale, the Beck Youth Inventory [39], which includes a sub-scale on self-concept, or the Harter Self-Perception Profile [40]. Significant improvements in self-esteem or body satisfaction were reported in the LOOZIT study (global self-worth; 0.21, 95% CI 0.01 to 0.32, p<0.001 and body shape dissatisfaction; -0.56, 95% CI -0.74 to -0.38, p<0.001) and the MEND study (global self-esteem score increase; 0.3, 95% C.I. 0.0 to 0.5, p = 0.03) [23, 27]. However, even in the studies where such improvements were not found, there were improvements to the children’s attitudes towards their health, which could have been facilitated by attempts to increase self-esteem.

Increasing self-efficacy to lead healthy lives was targeted specifically by the CHILDREN programme [20]. Self-efficacy was targeted through techniques such as modelling, enactment and guided practice in weekly sessions that lasted one to two hours [20]. This study did not, however, measure the change in self-efficacy, instead, change in diet, physical activity and sedentary behaviour were measured.

### Practical and cognitive tools

The practical and cognitive tools theme included components such as goal setting, where the children had to make goals for themselves and report back at a later time whether these were achieved or not [20, 22–26]. Where they were not achieved, discussions were held about the reasons why this may be and ways in which problems arising could be dealt with. Children were given practical skills such as how to read nutrition labels on food packaging, how to modify recipes to make them healthier, and how to make healthy choices, for example when eating at a restaurant [27, 32]. Cognitive skills, such as ways in which stress could be managed more effectively through improving the children’s communication skills, were also used by some studies [22–26].

All of the studies that aimed to provide children with practical or cognitive skills achieved improvements in either one or both of their physical activity levels and diet.

### Intervention characteristics

Unlike the preceding three themes, the intervention characteristics theme summarised the ways in which the successful interventions were delivered rather than what they delivered. Common characteristics included interventions that were interactive, the children were encouraged to discuss, play games and use role play to facilitate their learning [20], and that both children and those delivering the intervention were provided with materials such as workbooks [20, 22–26, 28–30, 32–34]. The children’s workbooks contained physiological and health information, activities, and space to enter their own thoughts that could be kept after the intervention had finished. Materials given to the researchers or teachers were often provided with the intention of keeping the delivery of the intervention consistent across lessons and were made up of instructions on delivering the intervention, reproducible materials for the children, and guidance on how the intervention should be assessed.

A common component was the involvement of parents, peers or the community so that general knowledge could be increased and support given to the children participating in the intervention [22–26, 28–30, 32, 37]. Finally, some of the studies used professionals, such as dieticians, in the design and delivery of the intervention [28–30, 32].

## Discussion

The purpose of this component analysis was to establish the components common to interventions in children that contribute to improvements in cardiovascular health parameters. The components we identified were classified into four themes: Health Behaviours, Self-Concept, Practical and Cognitive Tools, and Intervention Characteristics. To the best of our knowledge, this is the only study that has set out to determine which components are common to successful interventions to improve cardiovascular health in school aged children. Although the components of successful cardiovascular health promotion interventions have not been summarised previously, the themes that we have identified have been explored individually to a greater or lesser extent. A previous systematic review found inconsistent results when health behaviours were targeted to tackle obesity when only nine out of 20 interventions were able to show a significant improvement in mean BMI in the intervention group [41]. These papers were however seeking to change the BMI of their child participants, a goal that has been recognised to be difficult to achieve especially in the long term [42], and which was not the aim for this component analysis.

The Self Concept theme was created to encompass both self-esteem and self-efficacy. Self-efficacy forms a central part of Social Cognitive Theory, whereby the belief that an individual is able to act in order to bring about a desired change is central to them taking on that action [43]. As CVD is so strongly linked to behavioural factors, an individual’s self-efficacy to make lifestyle choices that are healthful rather than harmful, or modify their harmful behaviours is very important in preventing CVD.

The Practical and Cognitive Tools theme has been discussed previously in a 2011 paper by Lueke [44]. This paper advocates teaching children how to make healthy decisions and face the challenge of living healthily, rather than shielding them from unhealthy influences. The paper recommends that children are provided with such tools, even though some may argue that they have little control over their own diets, as they will be able to practice their newly gained skills while they are young and so will be prepared for when they have to make all their own decisions [44].

Finally the Intervention Characteristics theme suggests ways in which successful interventions should be carried out including the provision of student and teacher materials to ensure the faithful delivery of the intervention and the avoidance of type III errors. Type III errors have been defined as the attempt to assess the effectiveness of an intervention that has not been carried out in the way it has been designed, so that there is no testable relationship between the intervention and the factors it was designed to influence [45]. Therefore, by including instructions for teachers and materials for both teachers and students the risk of type III errors may be reduced.

### Strengths and weaknesses

The strength of this component analysis lies in the structured and systematic way in which relevant studies were identified for inclusion and the efforts made to ensure that their design was as comparable as possible. In this way 12 studies were selected and four component themes were identified that have been used to promote heart health to children and were successful at least in the short term. This component analysis is however not without its weaknesses. It is possible that our database search failed to identify some relevant studies that should have been included in the analysis. It is our belief however that as our searches were designed carefully and carried out in multiple databases this is unlikely. A second weakness is that although great care was taken to ensure that the included studies were as comparable and matched our requirements as closely as possible, there was still a level of heterogeneity among them in the ways in which they were delivered, their duration and the characteristics of the children to whom they were delivered. We believe however that as this analysis was descriptive rather than quantitative, this limitation is not serious enough to negate the findings.

### Implication for research and practice

The purpose of this study was to identify the components associated with education interventions that were successful in improving the health and health behaviours of children so that they might be incorporated into new interventions to target childhood cardiovascular health. It was found that an emphasis should be placed on how behaviours impact on internal mechanisms so that why healthy living is important might be highlighted to children. Second, the self-esteem of the child should always be central to any intervention and interventions should be designed so that weight bias is not perpetuated, but instead children are encouraged to see themselves as able to influence their own health outcomes and are equipped with the confidence and self-efficacy to do so. Third, children should be given practical and cognitive tools by health lessons that will assist them in their health decision making. Such skills could include stress management, reading nutrition labels, understanding how diet and physical activity interact to either promote or impede health, adapting recipes, and communication skills. Finally, health lessons should be interactive and hands on with materials for both the student and the teachers delivering the lessons so that they may be delivered with fidelity to the intervention design, allowing the effectiveness of the intervention to be assessed appropriately.

## Conclusion

The component themes identified were Health Behaviours, Self-Concept, Practical and Cognitive Skills and Intervention Characteristics. We conclude that as these themes have arisen from intervention studies that have had success in improving the health and health behaviours of children, they should be incorporated into future studies with similar aims so that these studies might build on the knowledge gained by their predecessors.

## Supporting Information

S1 PRISMA Checklist (DOC)

S1 Table Outcomes in which a significant (p<0.05) improvement was seen compared to control. (DOCX)
