# Supplementary material for: Moving Focus from Weight to Health. What Are the Components Used in Interventions to Improve Cardiovascular Health in Children?
Source: PLoS One. 2015 Aug 11;10(8):e0135115. doi: 10.1371/journal.pone.0135115 (PMC4532360; doi:10.1371/journal.pone.0135115)
Supplement: S1 Table — (DOCX) [file pone.0135115.s002.docx]

| Author | Study name | BMI* | Weight* | Waist circumference | Systolic blood pressure | Diastolic blood pressure | HOMA-IR | Triglycerides | LDL Cholesterol | HDL Cholesterol | Total Cholesterol | Knowledge | Attitudes | Behaviour |
| --- | --- | --- | --- | --- | --- | --- | --- | --- | --- | --- | --- | --- | --- | --- |
| Taylor et al. (2007) [33 34] | APPLE | X | X | X |  |  |  |  |  |  |  |  |  | X some measures of dietary intake and physical activity |
| Angelopoulos et al. (2009) [20] | CHILDREN | X |  |  | X | X |  |  |  |  |  |  |  | X some measures of dietary intake and physical activity |
| Melnyk et al. (2009) [21] | COPE TEEN |  |  |  |  |  |  |  |  | X |  | X |  | X healthy lifestyle |
| Schofield et al. (2005) [38] | GSOP |  |  |  |  |  |  |  |  |  |  |  |  | X some physical activity measures |
| Stock et al. (2007) [35] | Healthy Buddies | X | X |  | X |  |  |  |  |  |  | X | X | X in older children and females |
| Johnson et al. (1991) [37] | Heart Smart Program |  |  |  |  |  |  |  |  |  |  |  |  | X some dietary intake measures and physical activity measure |
| Nguyen et al. (2012) [22-26] | LOOZIT | X |  |  |  |  |  | X |  |  | X |  |  | X screen time |
| Sacher et al. (2010) [27] | MEND | X |  | X | X |  |  |  |  |  |  |  |  | X some measures of physical activity |
| Bayne-Smith et al. (2004) [36] | PATH |  |  |  | X | X |  |  |  |  |  | X |  | X breakfast consumption |
| Nemet et al. (2005) [32] | - | X | X |  |  |  |  |  |  |  |  |  |  | X physical activity |
| Park et al. (2007) [31] | - | X | X | X | X |  | X | X | X |  | X |  |  |  |
| Tershakovec et al. (1998) [28-30] | - |  |  |  |  |  |  |  | X |  |  | X |  | X some dietary intake measures |

**S1 Table. Outcomes in which a significant (p<0.05) improvement was seen compared to control**

*BMI and weight improvements include improvements in BMI_z_ and weight z-scores
